# Evaluation of the prevalence of coronary artery disease in patients with valvular heart disease

**DOI:** 10.1186/s13019-014-0153-1

**Published:** 2014-09-02

**Authors:** Zeynep Yapan Emren, Sadık Volkan Emren, Barış Kılıçaslan, Hatice Solmaz, İbrahim Susam, Ahmet Sayın, Burçin Abud, Mehmet Aydın, Özgür Bayturan

**Affiliations:** Cardiology Department, Sandıklı State Hospital, Afyonkarahisar, Turkey; Cardiology Department, Izmir Katip Celebi University, Ataturk Research and Training Hospital, İzmir, Turkey; Cardiology Department, Izmir Tepecik Research and Training Hospital, İzmir, Turkey; Cardiovascular surgery Department, Izmir Tepecik Research and Training Hospital, İzmir, Turkey; Cardiology Department, Manisa Celal Bayar University, Medical Faculty Hafsa Sultan Research Hospital, Manisa, Turkey

**Keywords:** Mitral regurgitation, Aortic stenosis, Coronary artery disease

## Abstract

**Objectives:**

The aim of the present study was to retrospectively evaluate the prevalence of concurrent coronary artery disease in patients who underwent surgery due to severe valvular heart disease. The study also investigated the association of coronary artery disease with the type of valvular heart disease.

**Materials and methods:**

A total of 241 patients (123 females [51%]), who had underwent single valvular heart surgery, were included in the study. The patients who underwent valve replacement surgery were divided into four groups: patients with severe mitral stenosis (MS), patients with severe mitral regurgitation (MR), patients with severe aortic regurgitation (AR), and patients with severe aortic stenosis (AS). Age, DM, HT, history of smoking, and LDL values were recorded as the risk factors for CAD.

**Results:**

Coronary artery disease was detected in 26.4% of patients with mitral stenosis and 57.7% of patients with aortic stenosis. Of the patients with mitral insufficiency, 41.9% had CAD, and 44.4% of the patients with aortic insufficiency had CAD.

**Conclusion:**

The comparison of MS and AS groups revealed significantly higher prevalence of CAD in the AS group. There was no statistically significant difference between the MR and AR groups in terms of the prevalence of CAD. The comparison of MS and MR groups revealed significantly higher prevalence of CAD in the MR group. Furthermore, the comparison of these groups in terms of the extensiveness of the coronary artery disease revealed significantly higher Gensini score in the MR group.

## Background

Currently, rheumatic factors in the etiology of valvular heart diseases have been mostly replaced by degenerative factors, the prevalence of which increase with increasing age [1]. In parallel to the change in the epidemiology of valvular heart disease, coronary artery disease (CAD) have become more commonly found in association with valvular heart disease particularly in the developed western countries [1]. The prevalence of degenerative aortic valve disease increases with age and it is the leading valvular condition accompanying CAD. Ischemic mitral valve insufficiency has become the focus of recent studies with regard to its diagnosis and treatment approaches [1].

The aim of the present study was to retrospectively evaluate the prevalence of concurrent coronary artery disease in patients who underwent surgery due to severe valvular heart disease and to investigate the association of coronary artery disease with the type of valvular heart disease.

## Methods

### Patient group

The medical records of 241 patients, who underwent valvular heart surgery in the Department of Cardiovascular Surgery in three different centers (Celal Bayar University Hafsa Sultan Hospital, Tepecik Training and Research Hospital, and Katip Celebi University Ataturk Training and Research Hospital) between 2004 and 2012, were retrospectively reviewed. The results of coronary angiography performed before the operation and biochemical parameters were evaluated. The patients were divided into four groups: patients with severe mitral stenosis (MS), patients with severe mitral regurgitation (MR), patients with severe aortic regurgitation (AR), and patients with severe aortic stenosis (AS). In addition, cardiovascular risk factors of the patients (age, gender, hypertension, smoking, diabetes mellitus) were recorded. The study was approved by the ethics committee of the Celal Bayar University Faculty of Medicine.

The patients who underwent multiple valvular heart surgery, patients aged below 18 years, patients who did not undergo coronary angiography, patients with mitral regurgitation whose transthoracic echocardiographic findings suggestive of ischemic etiology (ischemic left ventricular dysfunction, regional dysfunction of the LV myocardium, eccentric MY vs.), patients with mild and/or moderate valvular heart disease, re-operated cases due to prosthesis valve dysfunction, those with a previous history of valvular repair and balloon valvuloplasty were excluded from the study.

### Coronary angiography

The coronary angiography results were considered normal in the absence of plaques, irregular contour, ectasia, and slow flow in all epicardial coronary arteries (including lateral branches); however, coronary artery disease was defined as the presence of at least one of the above-stated conditions. The Gensini scoring system was used to evaluate the severity of CAD [2]. According to this scoring system, angiographic severity of the lesion was rated as follows: 1 point = 0-25%, 2 points = 25-50%, 4 points = 50-75%, 8 points = 75-90%, 16 points = 90-99%, and 32 points = 100% completely occluded vessel. The severity score was multiplied by the segment location multiplying factor (left main coronary artery lesion = 5 points, proximal left descending branch and left circumflex artery lesion = 2.5 points, middle left descending artery lesion = 1.5 points, first diagonal branch and the branches of obtus marginalis, and right coronary artery lesion = 1 point, and second diagonal and left circumflex artery posterolateral branch lesion = 0.5 points), and the results were summed to yield the Gensini score for each patient group.

### Statistical analysis

SPSS for Windows IBM SPSS Statistics 20 software package was used in the statistical analyses. The continuous variables were expressed as mean ± standard deviation, and non-continuous variables were expressed as proportion. The Student's t-test was used in the comparison of quantitative data with normal distribution, and chi-square test was used in the comparison of qualitative data. One-way ANOVA test was used in the comparison of multiple groups. The Kolmogorov-Smirnov test was used to test whether the variables were normally distributed. The Pearson's correlation coefficient was used in the correlation analysis. P values <0.05 were considered statistically significant.

## Results

A total of 241 patients (123 females [51%]), who underwent valvular heart disease, were included in the study. The study included 72 patients (29.9%) who underwent MVR due to severe mitral stenosis, 93 patients (38.6%) who underwent MVR due to severe mitral regurgitation, 40 patients (16.6%) who underwent AVR due to severe aortic stenosis, and 36 patients (14.9%) who underwent AVR due to severe aortic regurgitation. The demographic features of the patients are presented in Table 1. The Gensini scores in patients with DM and HT were found to be higher compared to patients without DM or HT (p = 0.012 and 0.026, respectively).

**Table 1:** **The demographic features of the study patients**

	MS (MVR) (n = 72)	MR (MVR) (n = 93)	AS (AVR) (n = 40)	AR (AVR) (n = 36)	P value
Age	52.23 ± 10.05	56.62 ± 12.07	63.52 ± 13.36	55.19 ± 14.21	0.000
HT (%)	5 (15.6)	15 (46.9)	7 (21.9)	5 (15.6)	0.008
DM (%)	20 (18.7)	47 (43.9)	22 (20.6)	18 (16.8)	0.284
History of Smoking (%)	22 (27.5)	28 (35)	15 (18.8)	15 (18.8)	0.547
TG (mg/dL)	116.18 ± 65.85	147.84 ± 95.40	143.32 ± 95.17	147.38 ± 75.42	0.087
LDL (mg/dL)	114.25 ± 32.06	112.37 ± 37.05	122.87 ± 39.35	114.88 ± 31.36	0.468

HT, hypertension; DM, diabetes mellitus; TG, triglyceride; LDL, low-density lipoproteins; MS, mitral stenosis; MR, mitral regurgitation; MVR, mitral valve replacement; AS, aortic stenosis; AR, aortic regurgitation; AVR, aortic valve replacement.

The coronary angiography results of the patients are presented in Table 2. There was significant difference between the groups in terms of the presence of CAD (p = 0.01). The prevalence of CAD was higher in patients with AS when compared to patients with MS (p = 0.002). The prevalence of CAD was higher in patients with MR when compared to patients with MS (p = 0.02). Although the difference between the groups in terms of the Gensini score did not reach statistical significance, the patients with MR had significantly higher Gensini score compared to patients with MS (2.97 ± 8.75 versus 11.02 ± 25.26, p = 0.005). CABG surgery was performed in addition to valvular heart surgery in 25% of the patients in the AS group, 12.9% of the patients in MR group, 8.3% of the patients in MS group, and 16.7% of the patients in AR group.

**Table 2:** **The results of CAG in the study patients**

	MS (MVR) (n=72)	MR (MVR) (n=93)	AS (AVR) (n=40)	AR (AVR) (n=36)	P value
Presence of CAD*	19 (26.4%)	39 (42%)	23 (57.5%)	16 (44.4%)	0.01
Gensini score**	2.97±8.75	11.02±25.26	11.20±28.26	7.55±16.98	0.077
CABG (%)	6 (8.3%)	12 (12.9%)	10 (25%)	6 (16.7%)	0.102
LVEF (%)	54.93±7.45	50.29±12.05	53.12±10.30	50.25±13.07	0.03

CAG, Coronary artery angiography; MS, mitral stenosis; MR, mitral regurgitation; MVR, mitral valve replacement; AS, aortic stenosis; AR, aortic regurgitation; AVR, aortic valve replacement. CAD, Coronary artery disease; CABG, Coronary artery bypass graft; LVEF, Left ventricular ejection fraction.

*The prevalence of CAD was significantly higher in AS group compared to MS group (p=0.002). The prevalence of CAD was significantly higher in MR group compared to MS group (p=0.02).

**The Gensini score was significantly higher in MR group compared to MS group (p=0.005).

There was significant difference between the groups in terms of left ventricular ejection fraction (p = 0.03). In Pearson's correlation analysis, LDL value was found to be correlated with the Gensini score (R = 0.171, p = 0.008). There was no significant correlation between the Gensini score and fasting blood glucose level (p = 0.85). There was inverse correlation between LVEF and the Gensini score (p < 0,001 R = -0,373). There was no significant correlation between pulmonary hypertension and the Hensini score (p = 0.20).

## Discussion

The previous studies have reported various prevalence rates for coronary artery disease in patients with valvular heart disease [1],[3]-[12]. However, there is no study in the literature that compared the patients with valvular disease as separate groups. In the present study, the patients who underwent valve replacement surgery due to severe valvular heart disease were evaluated in four different groups: patients with severe mitral stenosis, patients with severe mitral regurgitation, patients with severe aortic regurgitation, and patients with severe aortic stenosis. There was significant difference in terms of the prevalence of CAD when the groups were compared with each other and as a whole. In the study by Bozbas et al., out of 346 patients who underwent surgery due to rheumatic heart disease, 218 (63%) underwent coronary angiography, and 18.8% of the patients were found to have CAD [1]. In the study by Chun et al., 82 patients with MS underwent CAG, and 21 patients (26%) were found to have CAD [7]. Similar studies on patients with aortic stenosis reported a 56% prevalence rate for CAD [8],[9]. In the study by Shaikh et al. that evaluated 144 patients who underwent replacement of mitral valve, aortic valve or both, there was evidence for severe CAD in 32.9% of the patients who underwent MVR, 31.9% of the patients who underwent AVR, and 25% of the patients who underwent the replacement of both valves [10]. In the present study, 26.4% of patients with MS, 42% of the patients with MR, 57.5% of the patients with AS, and 44.4% of the patients with AR had CAD, a finding similar to that reported in the literature.

The atherosclerosis plays a role in the etiology of aortic stenosis as it already does in the etiology of CAD. Age-related calcification of the aortic valve is the most common cause of AS in the adult population [11]. The presence of calcific valve disease is associated with 50% increased risk of cardiovascular death and myocardial infarction even in the absence of valvular obstruction [12]-[14]. Valvular calcification is mediated by proliferative and inflammatory processes including lipid deposition, up-regulation of angiotensin converting enzyme activity, and infiltration of macrophages and T-lymphocytes [11],[12],[15],[16]. The risk factors for the development of calcific AS are similar to those involved in the development of vascular atherosclerosis. These risk factors include increased LDL-cholesterol and lipoprotein a, diabetes mellitus, smoking history, and hypertension. The retrospective studies have demonstrated slower progression rates in calcific AS with the administration of statin therapy [17]-[19], and this effect was further confirmed in an animal model [20]. Therefore, there is a growing consensus that degenerative calcific AS shares common pathophysiological features and metabolic pathways with atherosclerosis that can be targeted to prevent or delay the progression of disease [13],[16],[21],[22].

Furthermore, CAD plays a role in the etiology of MR. The disorders of the left ventricular papillary muscle are common causes of MR due to the fact that these muscles are supplied by the terminal portion of the coronary vascular bed and particularly sensitive to ischemia. The impairment in the coronary perfusion may result in papillary muscle dysfunction. A transient ischemia results in a temporary papillary muscle dysfunction and temporary MR [23]. A sustained and severe ischemia in the papillary muscle results in papillary muscle dysfunction and scarring, and the development of chronic MR. The ischemic conditions more commonly affect the posterior papillary muscle compared to anterolateral papillary muscle. The ischemia in papillary muscle is mostly caused by coronary atherosclerosis. MR often develops in patients recovered from myocardial infarction [24],[25]. The most common cause of regurgitation is regional dysfunction in the left ventricular myocardium at the base of the papillary muscle that results in incomplete leaflet coaptation. Ischemic left ventricular dysfunction and dilated cardiomyopathy are important etiological factors in the development of MR. The dilation of the left ventricle due to any reason including ischemia changes the spatial relation between the papillary muscles and chorda tendinea and therefore contributes to the development of MR [24]-[27]. A certain extent of MR is found in 30% of patients being considered for coronary artery bypass grafting. In these patients, MR is secondary to ischemic damage of the papillary muscles and/or enlargement of the mitral valve. MR develops in 20% of the patients following acute myocardial infarction, and the prognosis is poor in these patients even in the presence of mild MR [28],[29]. From this point of view, the present study investigated whether the prevalence of CAD significantly differed depending on the type of valvular heart disease. Different than the other studies, the present study compared all four groups in terms of the presence of CAD and found significant difference between the four groups. The prevalence of CAD was significantly higher in patients with AS when compared to patients with MS. The high prevalence of CAD in patients with AS reflects the role played by the atherosclerotic process in the etiology of AS. The prevalence of CAD was significantly higher in patients with MR when compared to patients with MS. The patients with MR are expected to have higher prevalence of CAD compared to patients with MS due to the fact that CAD plays a major in the etiology of MR.

In the study by Lacy et al., the patients were evaluated for the presence of concurrent CAD (50% or greater occlusion in at least one major coronary artery), they found that 31.3% of the patients with MS had CAD and 19% had occlusive CAD, and 36.3% of the patients with MR had CAD and 18% had occlusive CAD, and 58.9% of the patients with AS had CAD and 21% had occlusive CAD, and 36.6% of the patients with AR had CAD and 30% had occlusive CAD [30].

Sonmez et al. evaluated 760 patients who underwent valvular heart surgery, and they found occlusive CAD in 15.8% of the patients (p < 0.001), and the highest prevalence rate was noted in patients with AS (p < 0.05), a finding similar to that reported in the present study. The risk factor most commonly associated with CAD was the family history followed by DM, hyperlipidemia, HT, and smoking [31]. In the present study, DM had the most significant association with CAD.

The prevalence rate reported in the present study for CAD is in general similar to that reported in the literature. Although the patients with AR had higher prevalence of CAD compared to patients with MR, the Gensini score was found to be higher in patients with MR when compared to those with AR. The difference between the groups, however, did not reach statistical significance. The findings of the present study in terms risk factors for CAD are similar to those reported in the literature. The present study differ from the literature in that the patients in the present study were grouped based on the presence of severe valvular pathologies.

## Conclusion

In the present study there was significant difference between the groups in terms of the presence of CAD (p = 0.01). The prevalence of CAD was higher in patients with AS when compared to patients with MS (p = 0.002). The high prevalence of CAD in patients with AS reflects the role played by the atherosclerotic process in the etiology of AS. The prevalence of CAD was significantly higher in patients with MR when compared to patients with MS. The patients with MR are expected to have higher prevalence of CAD compared to patients with MS due to the fact that CAD plays a major in the etiology of MR. The findings of the present study in terms risk factors for CAD are similar to those reported in the literature.

## Abbreviations

AR: Aortic regurgitation
AS: Aortic stenosis
AVR: Aortic valve replacement
CAD: Coronary artery disease
CAG: Coronary angiography
DM: Diabetes mellitus
HT: Hypertension
LDL: Low density lipoprotein
LVEF: Left ventricul ejection fraction
MR: Mitral regurgitation
MS: Mitral stenosis
MVR: Mitral valve replacement
